# Multivariate analysis of body mass index management capabilities in senior patients with chronic heart failure in China: a cross-sectional study

**DOI:** 10.3389/fpubh.2026.1767531

**Published:** 2026-04-20

**Authors:** Ping Liao, Siyuan Feng, Wuzhi Li, Lili E

**Affiliations:** 1 Ma’anshan People's Hospital, Maanshan, Anhui, China; 2 Fifth People's Hospital of Shanghai, Fudan University, Shanghai, China

**Keywords:** BMI management, chronic heart failure, seniors, influencing factors, senior patients

## Abstract

**Objective:**

This study aims to examine the current status of body mass index (BMI) management capabilities among senior patients with chronic heart failure (CHF) in China, identify key influencing factors, and provide evidence-based recommendations for the development of targeted intervention strategies.

**Methods:**

The study enrolled 550 senior patients diagnosed with CHF. Participants were assessed using three instruments: a general information questionnaire, a CHF BMI management scale, and a chronic disease self-efficacy scale. Descriptive statistics, Pearson correlation analysis, univariate analysis, and multiple linear regression analysis were employed to evaluate the level of BMI management capabilities and to identify influencing factors.

**Results:**

The mean score on the CHF BMI management scale was 17.43 ± 9.21, indicating an overall low level of BMI management capability. The mean score on the chronic disease self-efficacy scale was 31.95 ± 8.97. A positive correlation was observed between the scores on the chronic disease self-efficacy scale and the CHF BMI management scale (*r* = 0.34, *p* < 0.01). Multiple linear regression analysis identified family attention to patients’ health status, chronic disease self-efficacy, the subtype of chronic heart failure (CHF), and the duration of the disease as significant determinants of BMI management capabilities (*p* < 0.01). Collectively, these variables accounted for 17.9% of the variance observed in BMI management capabilities. Notably, family attention to the patients’ health status was identified as the primary determinant of BMI management capabilities in this population.

**Conclusion:**

Senior patients with CHF demonstrate limited BMI management capabilities, influenced by multiple factors; with family attention to health status being the most critical influencing factor. Healthcare providers should develop and implement targeted interventions for BMI management informed by these key influencing factors to improve the clinical outcomes and the quality of life for senior CHF patients.

## 1 Introduction

Chronic heart failure (CHF) is characterized by a prolonged and refractory course, with a 5-year survival rate comparable to that of malignant tumors. Consequently, it is often referred to as the “cancer of cardiovascular diseases” (1). CHF is one of the most challenging conditions among cardiovascular diseases in our aging population, representing the advanced stage and ultimate manifestation of various cardiovascular diseases (2). As the population ages and medical technology advances, an increasing number of patients receive treatment and survive, thereby becoming potential candidates for CHF, which has led to a rising prevalence of the condition (3). The incidence, readmission, and mortality rates of CHF remain persistently high, making it a major public health concern (4). According to data from the World Health Organization (WHO), the prevalence of CHF among the global population aged 65 years and older is as high as 10–15%, and it exhibits an exponential increase with advancing age, imposing a substantial medical and economic burden on families and society (5).

In China, both the prevalence and incidence of CHF increased with age, with prevalence exceeding 10% in those aged 70 and above (6). Meanwhile, the incidence rates were 892 and 1,655 per 100,000 person–years among persons who were 65–79 and ≥80 years of age, respectively (7). CHF not only inflicts physical distress on patients, it can also sap a person’s enthusiasm for life and lead to a reduction in their functional capacity, which hampers their capabilities to relish those aspects of life that were once highly significant. Studies have identified multiple influencing factors for CHF, among which excessive volume overload, sarcopenia, and abnormal body fat distribution are key drivers of disease progression (8).

Reflecting overall body weight changes, BMI serves as a convenient and low-cost preliminary screening tool for weight status that can assist in evaluating an individual’s weight classification (underweight, normal weight, overweight). BMI is included in clinical guidelines for heart failure management. Interestingly, excess weight and obesity are linked to better outcomes (9). However, while excess weight and obesity are surprisingly associated with a more favorable prognosis. This phenomenon, widely documented in numerous meta-analyses, is known as the obesity paradox (10). Notably, this paradox stands in stark contrast to the conventional understanding of obesity-related health risks in the general population. Its underlying mechanisms are associated with multiple factors among with multiple factors among senior patients with CHF, including nutritional reserves, muscle mass, and disease tolerance, rendering it a key point that cannot be overlooked in weight management and prognostic evaluation for this vulnerable population. As a core component of the comprehensive management of heart failure, BMI management directly impacts symptom control, exercise tolerance, and prognostic improvement in patients (11). BMI management capabilities refers to patients’ comprehensive capabilities to maintain an optimal BMI, control fluid retention, and optimize body composition through dietary regulation, fluid management, regular physical activity, and self-monitoring under the guidance of medical care. The level of these capabilities is closely correlated with readmission risk, quality of life, and long-term survival rate in CHF patients (12). A growing body of clinical research indicates that effective management of BMI can significantly reduce the incidence of volume overload-related complications, such as acute pulmonary edema and pleural effusion, in senior patients with CHF. Furthermore, it can enhance their cardiac function classification (13), and enhance patients’ self-care efficacy (14).

However, in China, the implementation of BMI management among the senior population encounters several challenges. These include age-related physiological declines, such as sarcopenia and cognitive impairment, the complexities associated with polypharmacy, and inadequate social support (15). Currently, most interventions targeting BMI management in senior patients with CHF primarily emphasize healthcare provider-led dietary and exercise guidance. However, there is often a lack of focus on assessing and enhancing patients’ self-management capabilities. This oversight can lead to poor compliance with intervention strategies and suboptimal long-term outcomes, which ultimately results in a low implementation rate of effective BMI management practices within this patient population. Enhancing self-management support could significantly improve adherence and outcomes (16). Although existing studies have acknowledged the significance of BMI management in patients with CHF, most of these investigations are limited to correlation analyses between BMI and clinical prognosis (17) with a paucity of systematic research focusing on BMI management capabilities in the CHF population. Therefore, it is imperative to clarify the current status and influencing factors of BMI management capabilities among senior CHF patients, and conduct a comprehensive exploration of their mastery of BMI management knowledge, implementation of self-management behaviors, barriers to management, and supportive care needs. This will provide a scientific evidence base for developing individualized and precise BMI management strategies.

This study aimed to investigate the current status of BMI management capabilities among senior patients with CHF and clarify the specific characteristics associated with BMI management in this population. By conducting an in-depth analysis of the factors influencing their BMI management, the study seeks to identify key determinants that affect these patients’ ability to manage their BMI effectively. A theoretical basis is provided for developing targeted BMI management interventions to improve compliance and effectiveness in senior patients with CHF, thereby enhancing their clinical prognosis and quality of life.

## 2 Methods

### 2.1 Study design

This study employed a cross-sectional design. Under predetermined inclusion and exclusion criteria, community-dwelling older adults were recruited via convenience sampling from the Ma’anshan People’s Hospital, China, between June 2024 and August 2025. Data were collected via face-to-face interviews using paper-based questionnaires. Each questionnaire was accompanied by an explanatory statement outlining the study objectives, emphasizing the voluntary nature of participation, and ensuring respondents that their data would be kept confidential.

### 2.2 Sample and sampling

Patients diagnosed with CHF who were admitted to Ma’anshan People’s Hospital were enrolled in this study. Inclusion criteria were as follows: ① Conforming to the diagnostic criteria for chronic heart failure ICD − 10 (18); ② Aged 60 years or older; ③ NYHA functional classification of II-IV; ④ Clear consciousness and the capabilities to communicate normally; ⑤ Voluntary participation in this study and provision of written informed consent while treated in hospital; ⑥ including but not limited to patients at a high risk of obesity. Exclusion criteria were as follows: ① Patients with mental disorders; ② unwilling to cooperate with the study; ③ with severe hearing or visual impairment; ④ with significant motor dysfunction; ⑤ complicated with malignant tumors, severe respiratory diseases, or uremia; ⑥ Patients with adverse events such as emergencies during hospitalization.

### 2.3 Instruments

#### 2.3.1 General information questionnaire

This instrument was specifically developed based on the clinical characteristics of CHF and aligned with research objectives, it incorporates sociodemographic variables and disease-related factors, including age, gender, educational level, marital status, living arrangement (whether living alone), family attention to the patient’s health, support from the workplace or community, CHF subtype, NYHA functional classification, and disease duration.

#### 2.3.2 BMI management scale

The scale demonstrated a Cronbach’s *α* coefficient of the scale was 0.843, the content validity index (CVI) was 0.88, and the test–retest reliability coefficient was 0.833. This instrument comprises 16 items across four dimensions: weight monitoring, BMI management knowledge, BMI management beliefs, and BMI management behaviors (19). A 3-point or 4-point Likert scale was employed for scoring, with higher scores indicating better BMI management capabilities in patients. The cultural validation and applicability of the BMI Management Scale among the Chinese population were examined (19). The Cronbach’s *α* coefficients for the individual dimensions ranged from 0.608 to 0.790.

#### 2.3.3 Chronic disease self-efficacy scale

This scale consists of 6 items and employs a 10-point Likert scoring method, with a total score ranging from 6 to 60. Higher scores indicate a higher level of self-efficacy in patients, reflecting greater confidence in disease management. Scores were categorized as follows: a score of < 36 denotes a low level, 36–47 indicates a moderate level, and a score of ≥ 48 represents a high level. The Cronbach’s *α* coefficient of this scale was 0.87 (20).

### 2.4 Data collection

The study employed a convenience sampling method alongside face-to-face interviews to gather data. Investigators provided standardized instructions to ensure participants understood the survey’s purpose and significance, addressing any questions and securing written informed consent prior to distributing the questionnaires. Participants were encouraged to fill out the scales independently; however, for those who required assistance, investigators helped complete the forms based on the participants’ verbal responses. All completed questionnaires were collected on-site and underwent manual screening to identify any incomplete forms, which were subsequently excluded from further analysis. According to the sample size calculation, the required sample size was no less than 10 times the number of items, with a 20% loss to follow-up rate considered. Given the strong cardiovascular specialty of our hospital and the adequate patient pool, the final target sample size was set at 500. Out of the 550 questionnaires distributed, 528 were deemed valid, resulting in an effective response rate of 96%. This high response rate indicates strong engagement and reliability in the collected data for the study.

### 2.5 Data analysis

A database was established using Microsoft Excel, with data entered independently by two researchers to ensure accuracy. Statistical analyses were performed using SPSS 22.0 software. Descriptive statistics were applied to participants’ general characteristics, BMI management capabilities, and chronic disease self-efficacy. Categorical data were presented as frequencies (n) and percentages (%) while continuous data were expressed as mean ± standard deviation (x ± s). Pearson correlation analysis was conducted to explore the association between BMI management capabilities and self-efficacy. Univariate analysis was used to identify variables with statistically significant correlations to BMI management capabilities. Multiple linear regression analysis was then performed, with BMI management capabilities as the dependent variable and the statistically significant variables identified in the univariate analysis as independent variables; model fit was further evaluated. A two-tailed significance level of *α* = 0.05 was set for all statistical tests.

## 3 Results

### 3.1 General characteristics of the study population

In this study, a cohort of 528 senior patients diagnosed with CHF was enrolled in this study, with ages ranging from 65 to 93 years (mean = 77.79 ± 6.90 years). Of these participants, 53.8% were male, and 94% had attained an educational level of junior high school or below. Furthermore, 78% of the patients were married, while 16.7% resided alone. The largest proportion of patients had a disease duration of less than 1 year (44.7%), while only a small minority received support from their work units or communities (2.3%). Notably, 50% of the patients reported that their family members attached great importance to their health status. In terms of clinical characteristics, 90.9% of the participants were diagnosed with biventricular heart failure, and 84.1% were classified as NYHA functional class IV (see Table 1).

**Table 1:** Comparison of BMI management capabilities in senior CHF patients with different characteristics.

Item	Category	Case number (*n*)	Constituent ratio (%)	Physical management capabilities score ( $\bar{x}\pm s$ )	Statistic	*p-*value
Sex	Male	284	53.8	17.54 ± 9.49	0.297^a^	0.767
	Female	244	46.2	17.30 ± 8.90		
Disease	<1 year	236	44.7	15.65 ± 8.12	11.43^b^	<0.001
	1–5 years	168	31.8	17.95 ± 9.35		
Course	6-10 years	100	18.9	18.68 ± 10.43		
	>10 years	24	4.6	26.08 ± 6.93		
Marital	Married	412	78.0	18.36 ± 9.60	9.977^b^	<0.001
	Unmarried	12	2.3	14.83 ± 6.83		
Status	Widowed	104	19.7	14.04 ± 6.72		
Living	Yes	88	16.7	16.16 ± 8.91	-1.42^a^	0.156
Alone	No	440	83.3	17.69 ± 9.26		
Cardiac function	Grade II	12	2.3	14.17 ± 9.86	3.252^b^	0.039
	Grade III	72	13.6	15.28 ± 8.92		
Classification	Grade IV	444	84.1	17.87 ± 9.19		
Age (years)	60–69	76	14.4	16.42 ± 9.43	3.790^b^	0.023
	70–79	216	40.9	18.75 ± 9.65		
	≥80	236	44.7	16.55 ± 8.60		
Educational level	Illiterate	136	25.8	16.46 ± 8.46	2.908^b^	0.021
	Primary school	224	42.4	17.20 ± 9.39		
	Junior high school	136	25.8	18.91 ± 9.94		
	Senior high school	20	3.7	13.80 ± 6.22		
	Junior college above	12	2.3	22.17 ± 5.54		
Chronic heart	Left heart failure	48	9.1	13.81 ± 8.98	-2.875^a^	0.004
Failure type	Whole heart failure	480	90.9	17.79 ± 9.16		
Family attention to patient's health	High attention	264	50.0	20.50 ± 9.30	32.894^b^	<0.001
	Moderate attention	248	47.0	14.32 ± 8.02		
	Neglect	16	3.0	15.00 ± 8.63		
Unit/community Support	None	516	97.7	17.40 ± 9.27	-0.850^a^	0.412
	Available	12	2.3	19.00 ± 6.38		
Self-efficacy level	High level	24	4.6	24.75 ± 7.76	14.497^b^	<0.001
	Moderate level	156	29.5	19.18 ± 9.59		
	Low level	348	65.9	16.14 ± 8.77		

^a^represents *t*; ^b^represents F.

### 3.2 BMI management capabilities of senior CHF patients

The mean score of BMI management capabilities among senior CHF patients was 17.43 ± 9.21, indicating an overall low level. In descending order of the average item scores across the four dimensions, the ranking was as follows: BMI management beliefs, BMI management behaviors, weight monitoring, and BMI management knowledge (see Table 2).

**Table 2:** BMI management capabilities of senior CHF patients.

Item	Score range of original scale	Actual score interval	Total Score mean ( $\bar{x}\pm s$ )	Item average score	Score ranking
Total score of BMI management capabilities	0–42	4–36	17.43 ± 9.21	1.09	
BMI monitoring dimension	0–6	0–6	2.16 ± 1.58	0.72	3
BMI management knowledge dimension	0–8	0–7	2.42 ± 2.24	0.61	4
BMI management belief dimension	0–12	0–12	5.98 ± 2.85	1.50	1
BMI management behavior dimension	0–15	1–13	6.86 ± 3.71	1.37	2

### 3.3 Pearson correlation analysis between BMI management capabilities and self-efficacy in senior CHF patients

The mean score of self-efficacy among senior CHF patients was 31.95 ± 8.97, representing a moderately low level, and it was positively correlated with BMI management capabilities (*r* = 0.34, *p* < 0.01).

### 3.4 Univariate analysis of BMI management capabilities in senior CHF patients

The univariate analysis revealed statistically significant differences in BMI management capabilities among patients based on disease duration, age, chronic heart failure subtype, family support, self-efficacy, marital status, cardiac function classification, and educational level (*p* < 0.05) (see Table 1).

### 3.5 Multivariate analysis of influencing factors for BMI management capabilities in senior CHF patients

In conducting a multiple linear regression analysis with BMI management capabilities as the dependent variable, and disease duration, age, chronic heart failure subtype, family attention to patients’ health, self-efficacy level, marital status, cardiac function classification, and educational level as independent variables, the analysis aims to identify the extent to which each of these factors contributes to variations in BMI management capabilities. The results showed that the regression equation was statistically significant (*F* = 15.365, *p* < 0.001). Specifically, disease duration (*β* = 0.190, *p* < 0.001), chronic heart failure subtype (*β* = 0.114, *p* = 0.005), family attention to patients’ health (*β* = 0.239, *p* < 0.001), and self-efficacy level (*β* = 0.116, *p* = 0.007) were significant predictors of BMI management capabilities. Collectively, these variables explained 17.9% of the total variance in BMI management capabilities among senior CHF patients, indicating a good model fit. Among these factors, family attention to patients’ health was identified as the primary influencing factor for BMI management capabilities in senior CHF patients (see Table 3).

**Table 3:** Multivariate analysis of influencing factors for BMI management capabilities in senior CHF patients.

Model	Unstandardized coefficients		Standardized coefficient (*β*)	*t*	Significance	VIF
	B	Std. error				
(Constant)	−4.238	4.166		−1.017	0.310	
Age	−0.035	0.551	−0.003	−0.064	0.949	1.147
Disease course	1.968	0.427	0.190	4.606	<0.001	1.094
Chronic heart	1.830	0.649	0.114	2.820	0.005	1.055
Failure type						
Family attention to patient’s health	3.958	0.712	0.239	5.555	<0.001	1.192
Self-efficacy level	1.867	0.684	0.116	2.731	0.007	1.163
Marital status	−1.776	0.493	−0.154	−3.599	<0.001	1.176
Cardiac function	0.818	0.867	0.039	0.994	0.346	1.106
Classification						
Educational level	0.015	0.407	0.001	0.036	0.972	1.070
	Adjusted *R*^2^				0.179	
	*F*				15.365	
	*P*				<0.001	

## 4 Discussion

### 4.1 BMI management capabilities status in senior CHF patients

In this study, the mean score for BMI management capabilities among senior patients with CHF was 17.43 ± 9.21, indicating an overall low level. The mean item score for the knowledge dimension of BMI management was the lowest, whereas that for the belief dimension was the highest—a finding consistent with the results reported by Alebna et al. (14) and Wang et al. (19). The underlying causes are analyzed as follows. Firstly, with advancing age, the cardiac structure and function of senior patients undergo progressive degenerative changes. Impaired blood supply leads to cerebral hypoperfusion and other complications, which subsequently contribute to cognitive decline (21). Secondly, senior patients frequently present with multiple comorbidities, such as hypertension and diabetes mellitus. These conditions not only impose a heavy physical burden on patients but also divert their attention from BMI management, which further impairs the acquisition and application of relevant knowledge (22). Lastly, in China, senior patients are generally less receptive to new knowledge due to advanced age and other factors. Consequently, healthcare providers tend to prioritize delivering health guidance to the patients’ family members, which is a key factor contributing to the suboptimal acquisition of BMI management knowledge among senior patients. This phenomenon indicates that, when implementing health education initiatives, Chinese healthcare professionals should refrain from neglecting direct education for patients and instead adopt targeted strategies to enhance patients’ self-management capacity for their conditions.

Despite the patients’ deficiencies in mastering and comprehensively understanding BMI management knowledge, their scores in the belief dimension were relatively high. This indicates that they generally recognized the importance of BMI management, firmly believed that their condition could be alleviated through personal efforts, and were capable of implementing certain BMI management behaviors. Studies have shown that direct patient education helps patients gain insight into disease-related progression, enhances their attention to their own condition, and yields significantly more pronounced effects than family-focused education (23). Studies have demonstrated that, in light of the age-related cognitive decline among senior patients, designing simplified, easy-to-understand educational materials and delivering health guidance through illustrated, accessible formats can enhance knowledge acceptance capabilities. Establishing a multidimensional, multidisciplinary BMI management model involving the collaborative participation of patients, their family members, and healthcare providers is conducive to reinforcing the effectiveness of health education (24). In future endeavors, it is imperative to develop a patient-centered health education model. Utilizing formats such as family conferences and remote follow-ups, can ensure that patients and relevant stakeholders systematically acquire comprehensive knowledge of BMI management. Simultaneously, the incorporation of smart devices—including BMI scales and mobile applications—can support patients in conducting daily monitoring, thereby enhancing the convenience and sustainability of their self-management practices.

In the constructed multivariate regression model, independent variables explained 17.9% of the total variance in body weight management competence among old CHF patients, with a modest adjusted R-squared value. On the one hand, this low adjusted *R*^2^ indicates that the four incorporated factors—family health awareness, self-efficacy, CHF subtype, and disease duration—only account for a limited proportion of variance, suggesting the complexity of the underlying factor system that cannot be solely delineated by these four dimensions. On the other hand, this finding does not diminish the significance of the identified predictors; instead, it corroborates that body weight management ability in body weight management ability in senior CHF patients is jointly shaped by multi-dimensional factors, including physiological, psychological, familial, social, and disease-related aspects. A single dimension or a small set of factors is insufficient to fully elucidate its variation patterns. Moreover, these results offer valuable directions for future research, implying that subsequent studies can further explore potential determinants such as social support level, socioeconomic status, frequency of medical-nursing interventions, disease knowledge mastery, and caregiver competence to refine the predictive model and enhance its overall explanatory power.

### 4.2 The effect of chronic heart failure subtypes and disease duration on BMI management

This study demonstrated that patients with CHF and a disease duration of ≤1 year exhibited significantly lower BMI management efficacy (*p* < 0.05). Firstly, in the early stage of left-sided heart failure, pathological alterations are confined to left ventricular dysfunction, with relatively subtle clinical manifestations. Accordingly, patients often lack sufficient awareness of the clinical necessity of BMI management, which contributes to suboptimal treatment adherence. A previous study evaluated Patient-Reported Compliance in 475 senior HF patients, and the analysis revealed that poor adherence to management regimens is prevalent in the HF population. Notably, compliance was lowest with respect to exercise and daily body weight monitoring (25). Secondly, patients with a greater disease-related symptom burden are more inclined to proactively pursue BMI-focused self-management strategies. With disease progression (duration ≥ 1 year), patients with left-sided heart failure gradually develop right ventricular involvement, accompanied by exacerbated fluid retention manifestations. This clinical trajectory motivates them to mitigate discomfort via targeted interventions—including daily weight monitoring, sodium restriction, and fluid restriction—thereby facilitating concomitant improvements in both their perceived importance of BMI management and their ability to implement related strategies. During the transition from isolated left-sided heart failure to biventricular heart failure, patients’ perception of their disease shifts from a subclinical or minimally symptomatic state to one characterized by prominent clinical manifestations, which in turn triggers the activation of sustained self-management practices (26). Thirdly, the BMI management efficacy of patients with CHF was found to be positively associated with the depth of disease-related knowledge and symptom severity, which is consistent with the theoretical underpinnings of the Symptom-Driven Self-Management Model. The intensity of patients’ symptom perception directly modulates the initiation and sustainability of health-promoting behaviors. Supporting this, relevant evidence demonstrates that the implementation of symptom-triggered remote arrhythmia monitoring—targeting patients’ perceived physical impairments—results in quantifiable improvements in patient-reported self-management outcomes (27). Furthermore, through home visits and other outreach initiatives, healthcare practitioners can deliver structured, continuous guidance to post-discharge patients and supervise their adherence to BMI management protocols, which contributes to improved treatment compliance (28). Studies have demonstrated that digital health interventions—designed to provide self-management guidance, track health-related metrics, and facilitate peer support for patients with CHF—can guide transitional care toward behavioral reinforcement. Such interventions support real-time monitoring and personalized feedback, integrate clinical and health resources, and thereby address the temporal and spatial constraints associated with traditional follow-up care (29). Therefore, healthcare providers may develop a dual-component “early warning-co gnitive enhancement” intervention targeting newly diagnosed CHF (disease duration ≤1 year). Specifically, visual aids (e.g., symptom progression diagrams) may be utilized to facilitate patients’ comprehension of the progression risk from isolated left-sided heart failure to biventricular heart failure. Concurrently, dynamic weight tracking—such as daily morning fasting weight measurements—may be incorporated to foster a cognitive link between CHF-related symptoms and weight fluctuations, thereby strengthening patients’ awareness of the clinical necessity of BMI management.

### 4.3 Self-efficacy is a significant positive predictor of BMI management capacity in the senior CHF population

This study demonstrated a positive correlation between self-efficacy and BMI management efficacy among senior patients with CHF (Pearson correlation coefficient *r* = 0.34; *p* < 0.001). Self-efficacy levels in the study cohort were moderately low—an observation attributed to multidimensional pathophysiological and psychosocial factors. This finding elucidates the role of self-efficacy as a key psychological determinant in driving health-promoting behaviors among this patient population (30)——Patients with high self-efficacy can not only confront challenges in BMI management more proactively but also maintain behavioral consistency when experiencing symptom fluctuations. First and foremost, this finding closely aligns with the core tenet of Bandura’s Social Cognitive Theory, which posits that self-efficacy shapes health-related behaviors by influencing motivation, cognition, and emotional regulation (31), this finding reinforces the broader relevance of self-efficacy in senior chronic disease management. Notably, the study population consisted of adults aged ≥60 years. Age-related declines in exercise tolerance—attributable to age-associated physiological deterioration, comorbidities, and polypharmacy burden—have collectively eroded patients’ perceived control over self-management practices (32). “Treatment fatigue” resulting from recurrent disease exacerbations further compromises patients’ psychological resilience, ultimately perpetuating a vicious cycle whereby low self-efficacy leads to poor treatment adherence, which in turn contributes to disease progression and ultimately results in a further reduction in self-efficacy.

However, self-efficacy is a clinically modifiable psychological construct. Interventions grounded in self-efficacy theory primarily target the cultivation of “perceived capabilities”: enhancing patients’ self-efficacy through the accumulation of small incremental successes; facilitating vicarious experiences via peer case sharing; and reinforcing positive health beliefs through specialized clinical feedback. In this context, the establishment of senior nurse-led specialized community chronic disease management clinics can markedly enhance the professional rigor of such interventions. A meta-analysis by Ghizzardi et al. (33) demonstrated that motivational interviewing (MI) yields meaningful effects on self-care outcomes: specifically, moderate effects on improving self-care confidence and self-care management, and large effects on enhancing self-care maintenance. Therefore, healthcare practitioners can employ MI techniques to elicit patients’ intrinsic motivation. In addition, guiding patients to independently establish health goals through directive questioning can improve adherence to BMI management among patients with heart failure.

### 4.4 Family emphasis on patients’ health status acts as the dominant determinant of BMI management

This study further demonstrates that family involvement in senior patients’ health management exerts a significant positive influence on their BMI management efficacy: the greater the attention family members devote to patients’ health and the more comprehensive the assistance and support they provide, the higher the level of patients’ BMI management efficacy. This observation aligns with the findings reported by Zhu et al. (34). To contextualize this observation, the management of CHF typically requires at least three distinct classes of medications, each with unique dosing schedules. Additionally, patients must adhere to sodium restriction, sustain weight management practices, monitor early warning signs of disease decompensation, and titrate diuretic dosages according to changes in clinical manifestations (35). Without sufficient family assistance and support, the majority of senior CHF patients may face challenges in independently adhering to these complex management regimens. Furthermore, the integration of family-centered social support is critical—when family members engage in the development and supervision of BMI management plans, they can strengthen patients’ self-efficacy via emotional support and behavioral synchronization. Guided by the Individual and Family Self-Management Theory (IFSMT), this study further demonstrates that self-regulation, rather than disease complexity, serves as a pivotal determinant of self-management outcomes in patients with heart failure (36). Therefore, healthcare practitioners should prioritize attention toward patients lacking family support, encourage and guide family members to engage in patients’ daily care, leverage the collaborative role of families in BMI management, and provide them with multidimensional support covering knowledge supplementation, emotional reassurance, material assistance, and medical security. In this way, patients’ social support network can be strengthened to facilitate the effective implementation of BMI management.

## 5 Limitations

Despite its valuable insights, this study is limited by its single-center design: data were collected from a single tertiary general hospital located in a single geographic region, a design that reduces sample representativeness and restricts the generalizability of the findings. Future studies should adopt a multicenter study design with expanded sample sizes to further clarify the characteristics of factors influencing BMI management efficacy, thereby providing more robust evidence to guide clinical practice in the management of senior patients with CHF.

## 6 Conclusion

This study demonstrates that senior patients with CHF exhibit suboptimal BMI management efficacy. Specifically, family-centered health engagement, self-efficacy, CHF clinical phenotypes, and disease duration collectively explain 17.9% of the total variance in BMI management efficacy among this population, with family-centered health engagement exerting the most prominent influence. Healthcare providers should therefore develop targeted, factor-tailored interventions to improve BMI management efficacy in senior CHF patients, thereby mitigating disease progression and enhancing patients’ quality of life.

### 6.1 Implication to community

The findings of this study provide empirical evidence for establishing a three-tiered hospital-community-family collaborative management system. Moving forward, an integrated hospital-community-family framework could be developed to enhance patients’ self-efficacy, with distinct responsibilities assigned to each party: ① Hospital level: Deliver standardized health education, such as distributing BMI Management Manuals. ② Community level: Undertake continuous follow-ups and behavioral monitoring. Hospitals should share patients’ baseline BMI management data with community health service centers, which will take over transitional care and provide home-based guidance. Community nurses will act as implementers, guiding patients to correctly use weight scales, record fluid intake and output, and promptly identify abnormal weight fluctuations during home visits. ③ Family level: Strengthen daily support through initiatives like Family BMI Management Training Camps. Families will serve as supervisors, improving patients’ treatment adherence by participating in dietary planning (e.g., using salt-restriction spoons) and assisting with regular weight tracking.

This tripartite collaboration between hospitals, communities, and families will facilitate the sustainable improvement of BMI management capabilities among senior CHF patients.

## Data Availability

The original contributions presented in the study are included in the article/supplementary material, further inquiries can be directed to the corresponding author.
